# Elevated Circulating IL-10 Producing Breg, but Not Regulatory B Cell Levels, Restrain Antibody-Mediated Rejection After Kidney Transplantation

**DOI:** 10.3389/fimmu.2020.627496

**Published:** 2021-01-28

**Authors:** Yongsheng Luo, Feifei Luo, Kuanxin Zhang, Shilei Wang, Haojie Zhang, Xianlei Yang, Wenjun Shang, Junxiang Wang, Zhigang Wang, Xinlu Pang, Yonghua Feng, Lei Liu, Hongchang Xie, Guiwen Feng, Jinfeng Li

**Affiliations:** ^1^ Kidney Transplantation Unit, The First Affiliated Hospital of Zhengzhou University, Zhengzhou, China; ^2^ Biotherapy Research Center, Fudan University, Shanghai, China; ^3^Department of Digestive Diseases, Huashan Hospital, Fudan University, Shanghai, China; ^4^ Department of Colorectal Surgery, The First Affiliated Hospital of Zhengzhou University, Zhengzhou, China

**Keywords:** Breg phenotyping, kidney transplantation, antibody-mediated rejection, homeostasis, dynamic

## Abstract

**Background:**

Antibody-mediated rejection (AMR) occupies a major position for chronic rejection after kidney transplantation. Regulatory B cell (Breg) has been reported to have an inhibitory immune function, which contributes to the resistance for AMR.

**Methods:**

A nested case–control study for nine healthy donors, 25 stable (ST) patients, and 18 AMR patients was performed to determine the type of Breg in maintaining immune tolerance and preventing AMR.

**Results:**

Compared to the ST group, circulating interleukin (IL)-10^+^ Bregs, but not Bregs, significantly decreased. The receiver operating characteristic (ROC) curve analysis revealed that rather than the circulating Bregs, decreased circulating IL-10^+^ Breg levels were positively associated with AMR. However, kidney B cell and IL-10 infiltration was significantly increased in the AMR group with high expression of C-X-C motif chemokine 13 (CXCL13). In addition, circulating IL-10^+^ Bregs, rather than Bregs, remained higher than those at pre-operation, during the 90-day post-operation in immune homeostasis.

**Conclusion:**

The circulating IL-10^+^ Breg levels are more appropriate measures for assessing the resistance of AMR after kidney transplantation.

## Introduction

Kidney transplantation is the best choice for the end-stage treatment of chronic kidney disease. At present, antibody-mediated rejection (AMR), which is characterized by the presence of a donor-specific antibody (DSA) directed to human leukocyte antigen (HLA), has emerged as the leading cause of chronic damage to the kidney graft, which largely limits the long-term survival of the graft. (1, 2). However, existing schemes have failed to achieve promising results in the clinical treatment of AMR (1, 3, 4). Thus, there is an urgent need to identify potential biomarkers and risk factors for AMR after kidney transplantation, which is critical for treatment and patient outcomes.

Accumulating evidence suggests that under inflammatory circumstances, B cells can differentiate into antibody-secreting plasmablasts and regulatory B cells (Bregs), which are a special B cell subset that secretes immunosuppressive cytokines, particularly interleukin (IL)-10. (1, 5–7). Thus, plasmablasts produce alloantibodies, resulting in AMR, while Bregs exhibit immunoregulatory functions and help induce immune homeostasis. Thus, Bregs have been reported to be closely associated with the resistance to AMR after kidney transplantation (1, 8, 9).

Although the distinct surface markers of Breg subsets have not yet been identified in humans (1), previous studies have reported four common subpopulations of Bregs: memory Bregs (mBregs) defined with the CD19^+^CD24^+^CD27^+^ (10), transitional Bregs (tBregs) defined with CD19^+^CD24^+^CD38^+^ (10, 11), IL-10-producing memory Bregs (IL-10^+^ mBregs) defined with CD19^+^CD24^+^CD27^+^IL-10^+^ (12), and IL-10-producing transitional Bregs (IL-10^+^ tBregs) defined with CD19^+^CD24^+^CD38^+^IL-10^+^ (13). Laguna-Goya et al. reported that the reduction in tBregs and IL-10^+^ tBregs is positively associated with acute rejection after kidney transplantation (14). However, Zhou et al. reported that mBregs, rather than tBregs, significantly decreased in five patients with acute rejection at post-liver transplantation (10). Furthermore, Shabir et al. reported that tBregs were not associated with kidney allograft rejection in three AMR patients (15). In light of these inconsistent results, there is a need to further clarify which Breg subpopulation plays a vital role in AMR patients after kidney transplantation.

In the present study, the proportions of four subpopulations of circulating Bregs were compared among the healthy, stable (ST), and AMR groups. Then, the biodistribution of B cell, IL-10, and chemokines in the kidney grafts of healthy, ST, and AMR patients were determined. Furthermore, the dynamics of the four subpopulations of circulating Bregs in ST patients for 90 days after surgery were investigated. These present results, together those reported by previous studies (1), suggest that circulating IL-10^+^ mBregs and IL-10^+^ tBregs are the leading Breg subpopulations that contribute to the resistance of AMR.

## Materials and Methods

### Patients and Groups

All patients provided a signed informed consent prior to the study. The present study was approved by the Ethics Committee of the First Affiliated Hospital of Zhengzhou University (2018-KY-72). The clinical trial registration number is ChiCTR1900022501.

These patients were divided into three groups: (1) healthy control group (healthy, n = 9): healthy kidney donors within 18–55 years old (free of disease), consisting of the siblings and spouses of the recipients; (2) recipients with stable graft function group (ST, n = 25): patients within 18–55 years old, whose kidney function was stable (free of infection or rejection before and after kidney transplantation) during the follow-up period (≥6 months); (3) recipients with AMR group (AMR, n = 18): patients within 18–55 years old, who were diagnosed with AMR. The kidney grafts of recipients with ST and AMR were obtained from healthy kidney donors. The differential diagnosis between the ST and AMR groups was confirmed by two kidney pathologists, according to the Banff 2017 consensus (3, 16).

### Peripheral Blood, Kidney Tissues, and Cell Samples

The peripheral blood samples from the healthy group were collected at pre-operation, and at 1, 7 and 14 days post-operation. Samples obtained from the ST group were collected at pre-operation, and at 1, 7, 14, 30, and 90 days post-operation. Samples obtained from the AMR group were collected when rejection occurred (before the anti-rejection therapy for AMR). Peripheral blood mononuclear cells (PBMCs) were isolated from 5 ml of blood by Ficoll-Paque gradient centrifugation and preserved with 10% dimethyl sulfoxide (Solarbio Inc., Beijing, China) in liquid nitrogen.

The core needle renal biopsy specimens obtained from the healthy group were collected before surgery for time-zero donor kidney biopsies. The core needle renal biopsy specimens obtained from the ST group were collected at 90 days post-operation after their kidney function stabilized due to the protocol biopsy. In light of the second puncture impairment of kidney function, all AMR patients refused the core needle renal biopsy after anti-rejection therapy for AMR. Thus, core needle renal biopsy specimens from the AMR group were only collected when rejection occurred (before the anti-rejection therapy for AMR). A total of three healthy donors, nine ST patients, and nine AMR patients provided a signed informed consent before the core needle renal biopsy. The remaining six healthy donors, 16 ST patients, and nine AMR patients refused the core needle renal biopsy due to fear of major bleeding in association with the biopsy. Single cells were isolated from the core needle renal biopsy specimens of three healthy donors, three ST patients, and three AMR patients, by combining mechanical dissociation with the enzymatic degradation of the extracellular matrix, which maintains the structural integrity of tissues (Multi Tissue Dissociation Kit 1; Miltenyi Biotec, Bergisch Gladbach, Germany). The kidney cells were preserved with 10% dimethyl sulfoxide (Solarbio Inc., Beijing, China) in liquid nitrogen, and the remaining core needle renal biopsy specimens were embedded in paraffin at 4°C.

### Flow Cytometry

Cells stored in liquid nitrogen were quickly thawed in a water bath set at 37°C for 3 min. A total of 2–3 × 10^6^ PBMCs were incubated with the Roswell Park Memorial Institute 1640 dilution-cell stimulation cocktail (500×), which comprised of the PMA (Phorbol 12-Myristate 13-Acetate), a calcium ionophore (Ionomycin), and protein transport inhibitors Brefeldin A and Monensin, (eBioscience, San Diego, CA), at 37°C with 5% CO_2_ for 5 h. Dead cells were excluded by staining with 7-Aminoactinomycin D (Thermo Fisher Scientific, Pittsburgh, PA, USA), and the surface markers of Bregs were stained with fluorochrome-labeled antibodies (Brilliant Violet 421™ anti-human CD19, phycoerythrin (PE)/Cy7 anti-human CD24, Alexa FluorR647 anti-human CD27, Alexa FluorR488 anti-human CD38, and Brilliant Violet 510™ anti-human CD45; the appropriate isotype controls: Brilliant Violet 421™ Mouse IgG1 κ Isotype Control, PE/Cy7 Mouse IgG2a *κ* Isotype Control, Alexa FluorR647 Mouse IgG1 *κ* Isotype Control, Alexa FluorR488 Mouse IgG1 *κ* Isotype Control, and Brilliant Violet 510™ Mouse IgG1 *κ* Isotype Control; Biolegend, San Diego, CA, USA) for 30 min at 4°C, with protection from light. For the intracellular staining, cells were stained with antibodies targeting the intracellular cytokines of Bregs (PE anti-human IL-10; the appropriate isotype controls: PE Rat IgG1 κ Isotype Control; Biolegend, San Diego, CA, USA) for 30 minutes at room temperature, with protection from light. The labeled cells were analyzed using the BD Biosciences Canto II instrument (BD Biosciences, USA). A total of 100,000 events were acquired in the lymphocyte gate. The data analysis was performed using FlowJo software (Tree Star, San Carlos, CA, USA).

### Immunohistochemistry

Immunohistochemistry staining of CD19, IL-10, and C-X-C motif chemokine 13 (CXCL13) in kidney tissues was performed, respectively. Briefly, 4 μm sections obtained from formalin-fixed, paraffin-embedded kidney tissue samples were incubated with 1:500-diluted anti-CD19, 1:1000-diluted anti-CXCL13 (rabbit anti-human; Abcam, Cambridge, UK), and 1:500-diluted anti-IL-10 (rabbit anti-human; Absin, Shanghai, China) primary antibodies overnight at 4°C, followed by Horseradish Peroxidase-conjugated 1:20,000-diluted goat anti-rabbit secondary antibody (Abcam, Cambridge, UK) for 1 h at room temperature and then 3,3′-diaminobenzidine for another 10 min. DAPI appears in blue. Immunohistochemistry images were acquired with an Aperio ScanScope AT Turbo (Aperio, Vista, CA). Numbers of CD19, IL-10, and CXCL13 positivities were scored as follows: 0 positivity, score = zero; 1–5 positivities, score = one; 6–10 positivities, score = two; 11–20 positivities, score = three; >20 positivities, score = four.

### Statistical Analysis

In the nested case–control study, receiver operating characteristic (ROC) curve analysis was performed by MedCalc v18.11.3. The remaining statistical analyses were performed using IBM SPSS 21.0. Normally distributed measurement data with a homogeneity of variance were expressed as mean ± standard deviation ($\bar{x}$ ± s) and analyzed by independent-sample t-test (between-group comparisons) or one-way analysis of variance (among-group comparisons). Measurement data that did not have a normal distribution or homogeneity of variance were expressed in median with interquartile range (IQR) and analyzed by Mann–Whitney U test (between-group comparisons) or the Kruskal–Wallis test (among-group comparisons). Count data were analyzed by χ^2^ test, a corrected χ^2^ test, or Fisher’s exact test, as needed. One-way repeated measures analysis of variance was used at various time points. *P*-values <0.05 were considered statistically significant.

## Results

### Baseline Characteristics of the Patients

A total of nine healthy donors, 25 ST patients, and 18 AMR patients were included in the present study. As shown in **Table 1**, the gender of the recipients, age, body mass index (BMI), initial nephropathy, HLA mismatch, CNIs, warm-ischemia time, and cold-ischemia time did not differ among groups, or between groups (*P* > 0.05, for all). However, the incidence of both anti-class I and anti-class II DSA-positivity in the AMR group was significantly higher, when compared to those in the ST group (*P* < 0.001 for anti-class I DSA, *P* < 0.001; *P* = 0.001 for anti-class II DSA). Furthermore, compared with ST patients, AMR patients had significantly higher scores in tubulitis (t), interstitial inflammation (i), and peritubular capillaritis (ptc) (*P* = 0.012 for t; *P* < 0.001 for i and ptc), according to the Banff classification (normal, score = 0; mild, score = 1; moderate, score = 2; severe, score = 3) (3, 16) (**Supplemental Tables 1** and **2**).

**Table 1:** Characteristics of patients in the healthy, stable (ST) and antibody-mediated rejection (AMR) groups.

	Healthy	ST	AMR	P-value^*^
Total number (n)	9	25	18	–
Female/Male (n)	5/4	5/20	8/10	0.088
Age	42.11 ± 11.22	38.52 ± 9.17	33.44 ± 8.80	0.065
BMI (kg/m^2^)	23.25 ± 2.84	20.95 ± 2.57	20.94 ± 2.51	0.063
Initial nephropathy (n)^†^	–	13/5/3/4	8/2/0/8	0.120
Glomerulonephritis	–	13	8	–
IgA nephropathy	–	5	2	–
Hypertensive nephropathy	–	3	0	–
Other	–	4	8	–
HLA mismatch (n)^†^	–	3 (3, 3)	3 (3, 3)	0.749
Warm ischemic time (min)^†^	–	5 (4, 6)	6 (5, 8)	0.082
Cold ischemic time (h)^†^	–	7 (6, 8)	8 (7, 9)	0.116
CNIs (n)^†^	–	17/8	11/7	0.640
Tacrolimus	–	17	11	–
Cyclosporine A	–	8	7	–

ST, stable; AMR, antibody-mediated rejection; BMI, body mass index; HLA, human leukocyte antigen; CNIs, calcineurin inhibitors; Normally distributed measurement data with homogeneity of variance were expressed as the mean ± standard deviation and analyzed by one-way analysis of variance (among-group comparisons). Measurement data that did not have a normal distribution or homogeneity of variance were expressed as the median with interquartile range and analyzed by the Mann–Whitney U test (between-group comparisons). Count data were analyzed by the χ^2^ test. † The comparison between the ST and AMR groups. ^*^P-values <0.05 were considered statistically significant.

### Antibody-Mediated Rejection Patients Exhibited Decreased Circulating Interleukin-10^+^ Regulatory B Cell Levels

**Figure 1:**
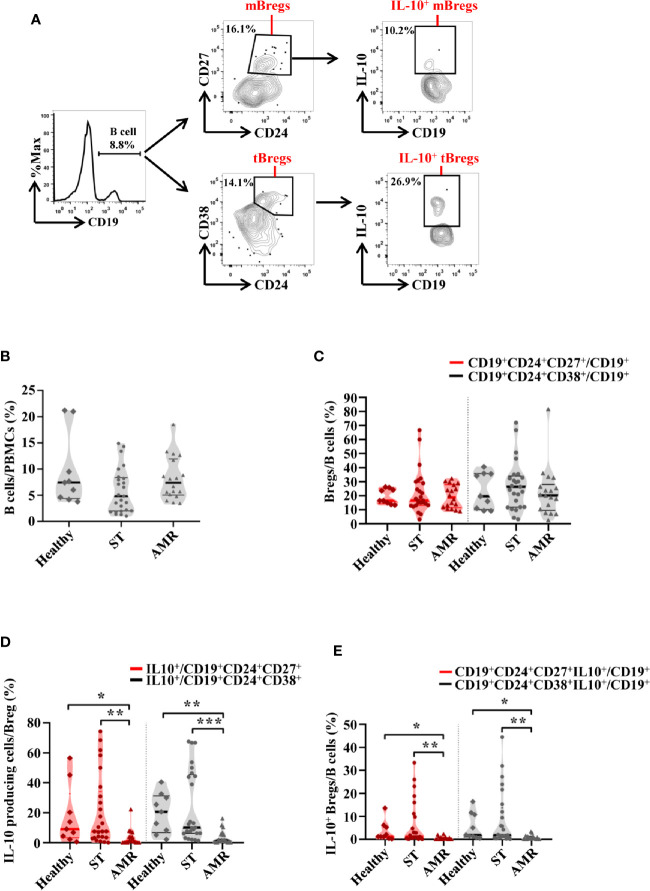
The percentages of four subpopulations of circulating Bregs among the healthy, stable (ST) and antibody-mediated rejection (AMR) groups. Flow cytometry analysis of peripheral blood mononuclear cells (PBMCs) isolated from healthy donors (n = 9) at pre-operation, the ST group (n = 25) at day 90 post-operation, and the AMR group (n = 18) at day 1 before anti-rejection therapy. **(A)** Representative dot plots depict the gating strategy to determine B cell subsets in PBMC. CD19^+^ B cells were identified based on high expression of CD19. Memory Bregs (mBregs) were identified from CD19^+^ B cells based on the high expression of CD24 and positivity of CD27. IL-10-producing memory Bregs (IL-10^+^ mBregs) were further gated based on the high expression of IL-10. Transitional Bregs (tBregs) were identified based on high expression of CD38 and CD24. IL-10-producing transitional Bregs (IL-10^+^ tBregs) were further gated based on the high expression of IL-10. **(B–D)** The statistical summary for the percentages of CD19^+^ B cells in PBMCs **(B)**, CD19^+^CD24^+^CD27^+^ mBregs or CD19^+^CD24^+^CD38^+^ tBregs in CD19^+^ B cells **(C)**, CD19^+^CD24^+^CD27^+^IL-10^+^ or CD19^+^CD24^+^CD38^+^IL-10^+^ Bregs (IL-10^+^ mBregs or IL-10^+^ tBregs) in CD19^+^ B cells **(D)**. Differences were evaluated by Kruskal–Wallis one-way analysis of variance (k samples) and *post-hoc* tests with all pairwise. *P < 0.05; **P < 0.01; ***P < 0.001.

In order to determine which Breg subpopulation could contribute to the resistance of AMR, four subpopulations of circulating Bregs were analyzed, from the healthy group before surgery, from the ST group at 90 days post-operation, and from the AMR group before the anti-rejection therapy. The mBreg, tBreg, IL-10^+^ mBreg, and IL-10^+^ tBreg were distinguished by flow cytometry (**Figure 1A**).

As shown in **Figure 1B**, the percentage of B cells (CD19^+^) in PBMCs were similar in the healthy, ST, and AMR groups (*P* = 0.095). The percentage of both mBregs and tBregs in B cells did not differ among groups (*P* = 0.949 for mBregs; *P* = 0.506 for tBregs) (**Figure 1C**). Additionally, it was found that compared with the AMR group, the percentages of the IL-10-producing cells in either mBregs or tBregs were significantly increased in the ST group [*P* = 0.001 for mBregs; *P* < 0.001 for tBregs] and the healthy group [*P* = 0.029 for mBregs; *P* = 0.004 for tBregs] (**Figure 1D**). In line with these results, it was found that the IL-10^+^ mBreg or IL-10^+^ tBreg ratios in B cells were significantly lower in the AMR group, when compared to the ST group (*P* = 0.002 for IL-10^+^ mBregs; *P* = 0.001 for IL-10^+^ tBregs) and healthy group (*P* = 0.017 for IL-10^+^ mBregs; *P* = 0.013 for IL-10^+^ tBregs) (**Figure 1E**).

### Decreased Circulating IL-10^+^ Regulatory B Cell Levels Were Positively Associated With Antibody-Mediated Rejection

**Figure 2:**
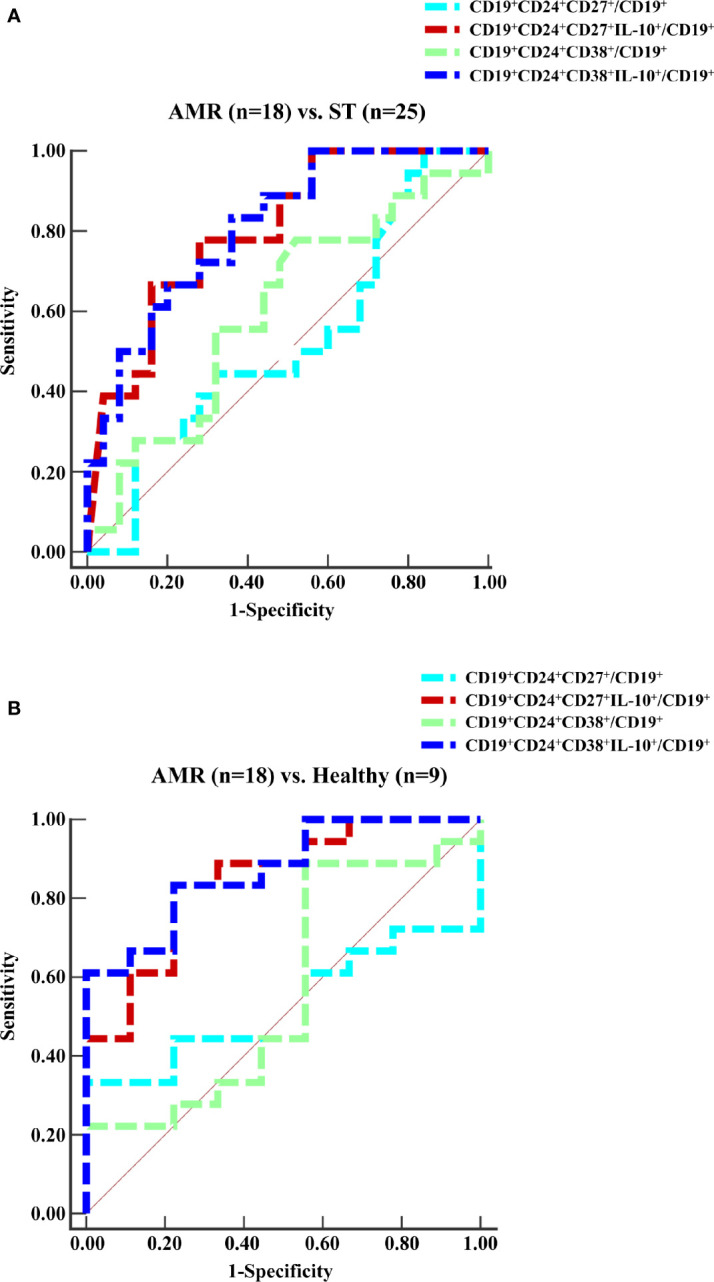
ROC curves for the ratios of four subpopulations of circulating Bregs. Peripheral blood mononuclear cells (PBMCs) were isolated from the ST group (n = 25) at day 90 post-operation, the healthy group (n = 9) before surgery, and the AMR group (n = 18) at day 1 before anti-rejection therapy. **(A, B)** The percentages of four subpopulations of circulating Bregs in B cells were analyzed by the ROC assay.

The diagnostic capacity of four subpopulations of circulating Bregs was compared between the ST (at day 90 post-operation) and AMR (before the anti-rejection therapy) groups by ROC curve analysis (**Figure 2A**). The diagnostic indicators are shown in **Table 2**. According to the criteria of Swets (18), although the mBreg or tBreg ratios in B cells had a low diagnostic accuracy, the IL-10^+^ mBreg or IL-10^+^ tBreg ratios in B cells exhibited a good sensitivity and area under the ROC curve (AUC) in diagnosing patients with AMR. Meanwhile, compared to the mBreg or tBreg ratios, the IL-10^+^ mBreg or IL-10^+^ tBreg ratios in B cells exhibited a statistically significant superior ability to discriminate between ST and AMR patients (*P* < 0.05 for all). Furthermore, similar results appeared between the healthy (before surgery) and AMR (before the anti-rejection therapy) groups (**Figure 2B** and **Table 3**).

**Table 2:** Comparison of different Breg subpopulations as a distinction between stable (ST) (n = 25) and antibody-mediated rejection (AMR) (n = 18) patients.

Factor	AUC(95% CI)	Threshold	Sensitivity(95% CI)	Specificity(95% CI)	PPV(95% CI)	NPV(95% CI)	LR+(95% CI)	LR–(95% CI)	DOR	*P*-values^*^
CD19^+^CD24^+^CD27^+^ /CD19^+^ (%)	52.8 (37.0–68.2)	11.8	27.8 (9.7–53.5)	88.0 (68.8–97.5)	62.5 (31.3–85.9)	62.9 (55.1–70.0)	2.3 (0.6–8.5)	0.8 (0.6–1.1)	2.8	0.004
CD19^+^CD24^+^CD27^+^IL-10^+^ /CD19^+^ (%)	80.3 (65.4–90.9)	0.5	66.7 (41.0–86.7)	84.0 (63.9–95.5)	75.0 (53.6–88.6)	77.8 (64.0–87.3)	4.2 (1.6–10.8)	0.4 (0.2–0.8)	10.4	
CD19^+^CD24^+^CD38^+^ /CD19^+^ (%)	60.6 (44.5–75.1)	20.5	55.6 (30.8–78.5)	68.0 (46.5–85.1)	55.6 (38.2–71.7)	68 (54.3–79.2)	1.7 (0.9–3.5)	0.7 (0.4–1.2)	2.7	0.008
CD19^+^CD24^+^CD38^+^IL-10^+^ /CD19^+^ (%)	81.1 (66.3–91.4)	0.2	50.0 (26.0–74.0)	92.0 (74.0–99.0)	81.8 (52.4–94.8)	71.9 (61.4–80.4)	6.3 (1.5–25.5)	0.5 (0.3–0.9)	11.6	

PBMCs, peripheral blood mononuclear cells; AUC, area under curve; CI, confidence interval; PPV, positive predictive value; NPV, negative predictive value; LR+, positive likelihood ratio; LR–, negative likelihood ratio; DOR, diagnostic odds ratio; ^*^P-values: DeLong et al. (17) for comparison of different receiver operating characteristic (ROC) curves. Obtained from ROC curves in ST vs. AMR patient samples.

**Table 3:** Comparison of different Breg subpopulations as a distinction between healthy (n = 9) and antibody-mediated rejection (AMR) (n = 18) patients.

Factor	AUC(95% CI)	Threshold	Sensitivity(95% CI)	Specificity(95% CI)	PPV(95% CI)	NPV(95% CI)	LR+(95% CI)	LR–(95% CI)	DOR	*P*-values^*^
CD19^+^CD24^+^CD27^+^ /CD19^+^ (%)	52.5 (32.5–71.9)	13.3	33.3 (13.3–59.0)	88.9 (51.8–99.7)	85.7 (45.8–97.7)	40.0 (30.9–49.9)	3.0 (0.9–21.3)	0.8 (0.5–1.1)	4.0	0.029
CD19^+^CD24^+^CD27^+^IL-10^+^ /CD19^+^ (%)	84.6 (65.5–95.5)	1.0	83.3 (58.6–96.4)	77.8 (40.0–97.2)	88.2 (68.5–96.3)	70.0 (44.0–87.4)	3.8 (1.1–13.0)	0.2 (0.1–0.6)	17.9	
CD19^+^CD24^+^CD38^+^ /CD19^+^ (%)	56.8 (36.5–75.6)	33.5	88.9 (65.3–98.6)	44.4 (13.7–78.8)	76.2 (63.6–85.4)	66.7 (30.9–89.9)	1.6 (0.9–2.9)	0.3 (0.1–1.1)	6.4	0.014
CD19^+^CD24^+^CD38^+^IL-10^+^ /CD19^+^ (%)	87.0 (68.5–96.8)	1.3	83.3 (58.6–96.4)	77.8 (40.0–97.2)	88.2 (68.5–96.3)	70.0 (44.0–87.4)	3.8 (1.1–13.0)	0.2 (0.1–0.6)	17.9	

PBMCs, peripheral blood mononuclear cells; AUC, area under curve; CI, confidence interval; PPV, positive predictive value; NPV, negative predictive value; LR+, positive likelihood ratio; LR–, negative likelihood ratio; DOR, diagnostic odds ratio; ^*^P-values: DeLong et al. (17) for comparison of different receiver operating characteristic (ROC) curves. Obtained from ROC curves in ST vs. AMR patient samples.

### Circulating IL-10^+^ Bregs Were Recruited to the Graft in Antibody-Mediated Rejection Patients

**Figure 3:**
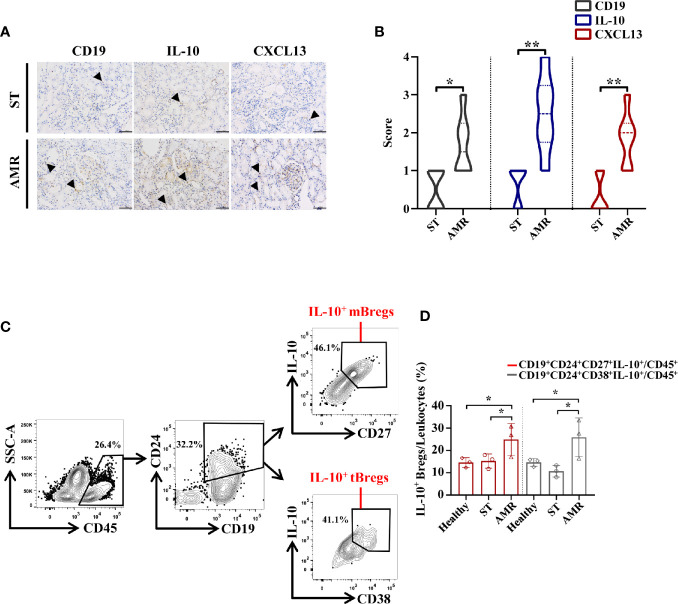
Analysis of IL-10^+^ Bregs in kidney tissue and cells from healthy, stable (ST) and antibody-mediated rejection (AMR) groups. **(A)** Immunohistochemistry was performed on kidney tissues from six ST patients and six AMR patients to detect CD19, IL-10, and CXCL13 (brown). DAPI staining (blue) was performed to visualize the nuclei. Scale bars, 100 μm. **(B)** The statistical summary for the scores according to the counts of CD19, IL-10, and CXCL13. **(C)** Kidney cells from three healthy donors, three ST patients, and three AMR patients were measured by flow cytometry. Representative dot plots depict the gating strategy to determine B cell subsets in kidney. CD45^+^ leukocytes were identified based on high expression of CD45. CD19^+^CD24^+^ cells were gated based on the high expression of CD24 and CD19. IL-10-producing memory Bregs (IL-10^+^ mBregs) were further gated based on the high expression of IL-10 and CD27 while IL-10-producing transitional Bregs (IL-10^+^ tBregs) were identified based on high expression of IL-10 and CD28. **(D)** The statistical summary for the percentages of CD19^+^CD24^+^CD27^+^IL-10^+^ or CD19^+^CD24^+^CD38^+^IL-10^+^ Bregs (IL-10^+^ mBregs or IL-10^+^ tBregs) in CD45^+^ leukocytes. The score differences were evaluated by Mann–Whitney U-test. The percentage differences were evaluated by one-way analysis of variance, and multiple comparisons were made with the least significant difference test. *P < 0.05; **P < 0.01.

The biodistribution of CD19, IL-10, and CXCL13 in grafted kidney tissues was analyzed through the immunohistochemistry staining in six ST and six AMR biopsy samples (**Figure 3A**). The mean count scores in B cells were significantly higher in the AMR group than in the ST group (*P* = 0.020). Then, it was found that the mean count scores in IL-10 positivity were significantly higher in the AMR group than in the ST group (*P* = 0.008). Additionally, compared with ST patients, AMR patients had significantly higher scores in CXCL13, which is a B cell specific chemokine (19) (*P* = 0.007) (**Figure 3B**).

Kidney cells were further collected from three healthy donors, three ST patients, and three AMR patients, respectively. After gating on CD45^+^ leukocytes (20, 21), the IL-10^+^ mBregs and IL-10^+^ tBregs were analyzed (**Figure 3C**). The percentages of IL-10^+^ mBregs or IL-10^+^ tBregs in CD45^+^ leukocytes were significantly higher in the AMR group than in the ST group (*P* = 0.044 for IL-10^+^ mBregs; *P* = 0.013 for IL-10^+^ tBregs) and healthy group (*P* = 0.035 for IL-10^+^ mBregs; *P* = 0.041 for IL-10^+^ tBregs) (**Figure 3D**).

### The Circulating Interleukin-10^+^ Regulatory B Cell Levels Remained High During Transplantation Homeostasis

The dynamic variation of four subpopulations of circulating Bregs in 25 ST patients was analyzed at 0, 1, 7, 14, 30, and 90 days post-operation. These four subpopulations of circulating Bregs were distinguished by flow cytometry (**Figure 1A**). Samples obtained from nine healthy donors were only collected at 0, 1, 7, and 14 days post-operation, respectively, due to shorter hospital stay. The mean percentage ± standard deviation and median with IQR at each time point in the ST group and healthy group are shown in **Supplemental Tables 3** and **4**.

**Figure 4:**
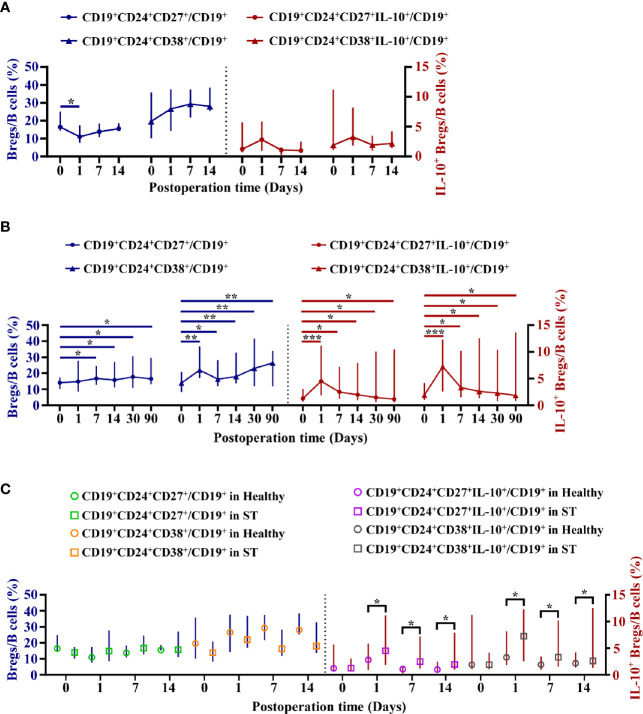
Dynamic variation of four subpopulations of circulating Bregs in healthy donors or stable (ST) patients. Flow cytometry analysis of peripheral blood mononuclear cells (PBMCs) isolated from nine healthy donors at 0, 1, 7, and 14 days post-operation and 25 ST patients at 0, 1, 7, 14, 30, and 90 days post-operation. **(A, B)** Dynamic variation of four subpopulations of circulating Breg ratios in Bcells in healthy donors **(A)** and ST patients **(B)**. **(C)** The differences of four subpopulations of circulating Breg ratios in Bcells between the healthy group and the ST group at the same time point. Comparisons between the healthy group and the ST group at the same time point were analyzed using independent-samples T test. Comparisons at different time points in healthy donors or ST patients were analyzed using one-way repeated measures analysis of variance, and multiple comparisons were made with the least significant difference test. *P < 0.05; **P < 0.01; ***P < 0.001.

In the healthy group, except that the mBreg ratios in B cells were lower at day one post-operation than those at pre-operation (*P* < 0.05), the four subpopulations of circulating Bregs had no significant differences between pre-operation and post-operation (*P* > 0.05) (**Figure 4A**). However, in the ST group, except that the mBreg ratios in B cells were non-significantly high at day one post-operation, compared to those at pre-operation (*P* > 0.05), the four subpopulations of circulating Bregs remained higher than those at pre-operation during the three months following transplantation (*P* < 0.05) (**Figure 4B**). Additionally, it was found that the mBreg or tBreg ratios in B cells had no significant differences at each time point between the healthy and ST groups (*P* > 0.05, for all). However, the percentage for both IL-10^+^ mBregs and IL-10^+^ tBregs in B cells were significantly higher at days 1, 7, and 14, post-operation, in the ST group, when compared to those in the healthy group (*P* < 0.05, for all) (**Figure 4C**). In line with these results, it was inferred that compared with the mBreg and tBreg ratios, the IL-10^+^ mBreg and IL-10^+^ tBreg ratios in B cells were more pivotal during the transplantation homeostasis.

## Discussion

Several studies have reported that Bregs can attenuate inflammation and contribute to the resistance of AMR (22–26). However, there is presently no comprehensive clinical research on the effect of the four subpopulations of Bregs in AMR patients after kidney transplantation. Thus, in the first step, the relationship between these cells and AMR occurrence was verified. The present flow cytometric analysis revealed that rather than the circulating mBregs and tBregs, the circulating IL-10^+^ mBregs and IL-10^+^ tBregs decreased in AMR patients, indicating that circulating IL-10^+^ Bregs are closely associated with AMR occurrence. The ROC curve analysis further demonstrated that compared with the circulating mBreg and tBreg levels, decreased circulating IL-10^+^ mBreg and IL-10^+^ tBreg levels were more suitable to use as a diagnostic tool for AMR. These results imply that IL-10^+^ mBregs and IL-10^+^ tBregs may play a vital role in AMR patients at post-kidney transplantation.

Subsequently, the immunohistochemistry assay revealed that the kidney B cell and IL-10 infiltration increased in AMR patients, when compared with ST patients. It was inferred that the increased B cells exhibit immunoregulatory functions in kidney grafts of AMR patients. Then, the flow cytometric analysis of kidney cells demonstrated that the kidney IL-10^+^ Breg ratios in leukocytes increased in AMR patients. In light of the contradictory results between peripheral blood and kidney grafts, a logical explanation was the recruitment of circulating IL-10^+^ Bregs to the graft. At present, the putative mechanisms leading to IL-10^+^ Breg infiltration in kidney tissues was largely attributable to chemokines, which has been generally considered to be the main mediators in leukocyte trafficking under inflammatory conditions (19, 27, 28). Indeed, it was also found that compared with ST patients, the expression of CXCL13 in kidney grafts was higher in AMR patients. These results were consistent with a previous report, in which CXCL13 and its receptor established distinct B cell rich compartments at the site of kidney tissue inflammation (19). Additionally, the present data revealed that compared with ST patients, both the IL-10 secreted by Bregs and the DSA sourced from plasmablasts increased in AMR patients with greater severity of interstitial and vascular rejection in kidney grafts. This was also in accordance with the report, in which there was a high prevalence of B cells in the interstitial rejection processes (19). Thus, it could be inferred that during AMR occurrence, the CXCL13 mediates the circulating IL-10^+^ Breg influx into kidney grafts, which leads to a large number of IL-10 production, subsequently contributing to the resistance of antibody-mediated interstitial and vascular rejection. On the contrary, since immune homeostasis has already been established, ST patients had less IL-10^+^ Breg influx into the kidney grafts. However, due to clinical technical problems that involve the tracing of IL-10^+^ Breg influx into kidney grafts, the inference needs to be further proven through experimental animal studies.

From another perspective, in order to further determine whether circulating Bregs also help in the maintenance of immune homeostasis, the dynamic variation of circulating Bregs in ST patients was analyzed. The present data revealed that only circulating IL10^+^ mBregs and IL10^+^ tBregs in the ST group at post-operation remained at a higher level, when compared to those in the healthy group, which had a higher level, when compared to those at pre-operation. Therefore, it was inferred that the high level of IL10^+^ Bregs may be a reserve force of anti-rejection, which can be mobilized to attenuate inflammation and resist rejection during AMR occurrence. Additionally, existing research has shown that the transfer of IL-10^+^ Bregs is obviously effective during AMR initiation (29, 30). Importantly, these findings provide an innovative perspective that the promotion of the circulating IL-10^+^ Bregs, such as the autologous transfer of IL-10^+^ Bregs during the early phase after kidney transplantation, may restrain the incidence of AMR.

One of the strengths of the present study is that this is a comprehensive kidney transplant cohort to prospectively measure four subpopulations of circulating Bregs to identify potential biomarkers and risk factors for AMR. Further studies using the circulating IL-10^+^ Bregs found in AMR, combined with other immunologic features, such as T cell phenotyping, observed in this state, will help to identify specific and sensitive parameters to evaluate the progression to AMR. The results of the present study are biologically plausible and extend the present literature, which supports efforts to further clarify IL-10^+^ Breg role in kidney transplantation, either as biomarkers of AMR risk, or in potential cell-based therapies. Certainly, the present study was limited by its small sample size, which impaired the ability to draw a definitive conclusion on the role of IL-10^+^ Bregs in AMR after kidney transplantation. Additionally, PBMCs from the AMR group should also be collected after the anti-rejection therapy.

## Data Availability Statement

The raw data supporting the conclusions of this article will be made available by the authors, without undue reservation.

## Ethics Statement

The studies involving human participants were reviewed and approved by the Ethics Committee of the First Affiliated Hospital of Zhengzhou University (2018-KY-72). The clinical trial registration number is ChiCTR1900022501. The patients/participants provided their written informed consent to participate in this study.

## Author Contributions

YL, FL, and KZ performed the experiments, analyzed the data, and wrote the manuscript. SW and HZ participated in the study design, material support, and coordination. XY, WS, JW, ZW, XP, YF, LL, and HX carried out parts of the experiments. JL and GF conceived the study and performed critical revisions of the manuscript. All authors contributed to the article and approved the submitted version.

## Funding

This work was supported by the National Natural Science Foundation of China (No. 82070771) and Foundation of Henan Provincial Health Bureau (SBGJ2018022).

## Conflict of Interest

The authors declare that the research was conducted in the absence of any commercial or financial relationships that could be construed as a potential conflict of interest.

The reviewer CY declared a shared affiliation, though no other collaboration, with one of the authors FL to the handling editor.
