# Supplementary material for: Elevated Circulating IL-10 Producing Breg, but Not Regulatory B Cell Levels, Restrain Antibody-Mediated Rejection After Kidney Transplantation
Source: Front Immunol. 2021 Jan 28;11:627496. doi: 10.3389/fimmu.2020.627496 (PMC7877339; doi:10.3389/fimmu.2020.627496)
Supplement: Supplementary file 1 [file Table_1.docx]

**Supplemental Table 1: Immunological characteristics of stable (ST) patients (n = 25).**

| Patient | Banff Classification | | | | C4d | DSA | |
| --- | --- | --- | --- | --- | --- | --- | --- |
|  | glomerulus | Renal tubule | Renal  interstitium | Renal  vessels |  | class I | class II |
| ST1 | g0mm0 | t1ct1 | i1ci0 | ptc0v0ah1 | Pos | – | – |
| ST2 | g2 | t1ct1 | i1ci0 | ptc0v0 | Neg | – | – |
| ST3 | – | t1ct1 | i1ci1 | ptc0v0 | Neg | Neg | Neg |
| ST4 | g1mm1 | t0ct0 | i0ci0 | ptc0v0 | Neg | – | – |
| ST5 | mm1 | t0ct0 | i0ci0 | v0 | Neg | – | – |
| ST6 | mm0 | t0ct1 | i1ci1 | v0 | Neg | – | – |
| ST7 | g1mm0 | t1ct0 | i1ci0 | ptc1v0 | Neg | – | – |
| ST8 | g0mm1 | t0ct0 | i1ci0 | ptc0v0ah1 | Neg | – | – |
| ST9 | g0mm0 | t3ct1 | i1ci1 | ptc0v0ah1 | Pos | – | – |
| ST10 | g0mm1 | t0ct1 | i1ci1 | ptc1v0ah1 | Pos | – | – |
| ST11 | g1mm1 | t0ct1 | i1ci1 | ptc0v0ah1 | Pos | – | – |
| ST12 | g1mm1 | t1ct1 | i1ci1 | ptc1v0ah1 | Neg | – | – |
| ST13 | g0mm0 | t0ct1 | i0ci2 | ptc0v1ah0 | Pos | Neg | Neg |
| ST14 | g1mm1 | t0ct0 | i1ci0 | ptc1v0 | Neg | – | – |
| ST15 | g1mm1 | t1ct1 | i1ci1 | ptc0v0ah1 | Neg | Neg | DQ4 (1353) |
| ST16 | mm1 | t0ct1 | i0ci0 | ptc0v0ah1 | Pos | – | – |
| ST17 | g0mm0 | t1ct0 | i1ci0 | ptc1v0ah1 | Neg | – | – |
| ST18 | g0 | t1ct1 | i1ci1 | ptc1v0ah1 | Pos | Neg | – |
| ST19 | g0mm1 | t0ct0 | i1ci0 | ptc0v0ah1 | Pos | – | – |
| ST20 | g0mm1 | t1ct1 | i1ci1 | ptc1v0ah2 | Pos | – | – |
| ST21 | g1mm1 | t1ct1 | i1ci0 | ptc0v0ah1 | Pos | – | – |
| ST22 | g0mm1 | t0ct0 | i1ci1 | ptc0v0 | Pos | Neg | Neg |
| ST23 | – | t0ct0 | i0ci0 | ptc0v0ah1 | Pos | Neg | Neg |
| ST24 | g1mm1 | t1 | i1ci0 | ptc1v0ah1 | Neg | – | – |
| ST25 | g0mm0 | t1ct1 | i1ci0 | ptc1v0 | Neg | – | – |

Abbreviations: ST, stable; DSA, donor-specific antibody; g, glomerulitis; mm, mesenchymal matrix hyperplasia; t, tubulitis; ct, atrophy of tubule; i, interstitial inflammation; ci, interstitial fibrosis; ptc, peritubular capillaritis; v, intimal arteritis; ah, arterial hyalinosis; Neg, negative; Pos, positive.
