# Supplementary material for: Elevated Circulating IL-10 Producing Breg, but Not Regulatory B Cell Levels, Restrain Antibody-Mediated Rejection After Kidney Transplantation
Source: Front Immunol. 2021 Jan 28;11:627496. doi: 10.3389/fimmu.2020.627496 (PMC7877339; doi:10.3389/fimmu.2020.627496)
Supplement: Supplementary file 2 [file Table_2.docx]

**Supplemental Table 2: Immunological characteristics of patients with antibody–mediated rejection (AMR) (n = 18).**

| Patient | Banff Classification | | | | C4d | DSA | |
| --- | --- | --- | --- | --- | --- | --- | --- |
|  | glomerulus | Renal tubule | Renal  interstitium | Renal  vessels |  | class I | class II |
| AMR 1 | g0mm0 | t3ct1 | i3 | ptc2v0 | Pos | A2 (1000) | DQ2 (6496) |
| AMR 2 | mm0 | t1ct2 | i1ci2 | ptc2v0 | Pos | – | DQ7 (24138) |
| AMR 3 | g1 | t3ct1 | i2ci0 | ptc1v0 | Neg | – | DQ4 (11879) |
| AMR 4 | g1 | t1ct2 | i2ci2 | ptc2ah2 | Pos | A34 (24084) | DR15 (8195) |
| AMR 5 | g1 | t1ct0 | i1ci0 | ptc1v0ah1 | Neg | B60 (808) | DR15 (15807) |
| AMR 6 | g0mm0 | t2ct0 | i2ci1 | ptc1v0ah1 | Pos | B44 (1889) | DQ7 (1232) |
| AMR 7 | g0mm1 | t0ct0 | i1 | ptc2v0ah1 | Pos | B48 (979) | – |
| AMR 8 | g1 | t1ct1 | i2ci2 | ptc2ah1 | Pos | A1 (6712) | – |
| AMR 9 | g0mm1 | t2ct1 | i2ci1 | ptc1v0 | Pos | A33 (407) | DQ7 (3103) |
| AMR 10 | g0 | t3ct2 | i2ci1 | ptc2v0 | Pos | B76 (6327) | DQ2 (24723) |
| AMR 11 | g1 | t0ct0 | i1ci1 | ptc1ah2 | Pos | – | DQ6 (21592) |
| AMR 12 | g1mm1 | t0ct1 | i1ci0 | ptc1v0ah1 | Pos | – | DR51 (20466) |
| AMR 13 | g1 | t1ct1 | i1ci1 | ptc2 | Pos | B77 (4161) | DQ8 (4633) |
| AMR 14 | – | t2ct0 | i2ci0 | ptc2v1 | Pos | A2 (4236) | DQ6 (13194) |
| AMR 15 | g0mm0 | t0ct0 | i1ci0 | ptc2v0 | Pos | A33 (7289) | Neg |
| AMR 16 | g2 | t1ct1 | i1ci0 | ptc3v0 | Pos | Neg | DQ7 (24536) |
| AMR 17 | g1 | t1ct1 | i1ci1 | ptc1 | Pos | A25 (1107) | DQ9 (11764) |
| AMR 18 | g0mm1 | t2ct0 | i2ci1 | ptc1v0ah1 | Pos | – | DQ7 (1370) |

Abbreviations: AMR, antibody–mediated rejection; DSA, donor-specific antibody; g, glomerulitis; mm, mesenchymal matrix hyperplasia; t, tubulitis; ct, atrophy of tubule; i, interstitial inflammation; ci, interstitial fibrosis; ptc, peritubular capillaritis; v, intimal arteritis; ah, arterial hyalinosis; Neg, negative; Pos, positive.
