# Supplementary material for: Elevated Circulating IL-10 Producing Breg, but Not Regulatory B Cell Levels, Restrain Antibody-Mediated Rejection After Kidney Transplantation
Source: Front Immunol. 2021 Jan 28;11:627496. doi: 10.3389/fimmu.2020.627496 (PMC7877339; doi:10.3389/fimmu.2020.627496)
Supplement: Supplementary file 3 [file Table_3.docx]

**Supplementary Table 3: The data of four subpopulations of circulating Bregs at each time point in stable (ST; n=25) patients.**

| ratio | statistic | day 0 | day 1 | day 7 | day 14 | day 30 | day 90 |
| --- | --- | --- | --- | --- | --- | --- | --- |
| CD19^+^CD24^+^CD27^+^  /CD19^+^ (%) | ‾x | 15.26 | 18.72 | 21.29 | 23.29 | 26.80 | 22.35 |
|  | s | 7.27 | 12.07 | 14.85 | 18.39 | 24.39 | 15.23 |
|  | M | 14.00 | 14.80 | 16.70 | 15.70 | 17.70 | 16.50 |
|  | IQR | 10.19–17.35 | 8.59–27.65 | 12.70–24.50 | 11.05–27.05 | 10.80–30.60 | 13.10–29.45 |
| CD19^+^CD24^+^CD38^+^  /CD19^+^ (%) | ‾x | 15.19 | 26.45 | 21.77 | 24.79 | 30.56 | 27.35 |
|  | s | 7.32 | 14.05 | 15.77 | 16.70 | 24.94 | 17.47 |
|  | M | 13.90 | 21.90 | 16.30 | 17.90 | 23.00 | 26.40 |
|  | IQR | 8.32–20.80 | 16.90–36.85 | 11.90–28.15 | 13.65–32.90 | 11.90–41.65 | 11.85–34.00 |
| CD19^+^CD24^+^CD27^+^IL-10^+^  /CD19^+^ (%) | ‾x | 2.03 | 6.83 | 6.00 | 8.00 | 12.07 | 6.45 |
|  | s | 2.18 | 6.60 | 9.00 | 13.52 | 25.11 | 9.51 |
|  | M | 1.25 | 4.51 | 2.47 | 1.97 | 1.46 | 1.14 |
|  | IQR | 0.67–3.06 | 1.88–11.11 | 1.17–7.17 | 0.96–7.90 | 0.57–10.04 | 0.55–10.49 |
| CD19^+^CD24^+^CD38^+^IL-10^+^  /CD19^+^ (%) | ‾x | 2.98 | 9.94 | 8.07 | 10.29 | 13.87 | 8.64 |
|  | s | 2.92 | 9.64 | 12.08 | 16.08 | 26.49 | 12.05 |
|  | M | 1.90 | 7.24 | 3.30 | 2.59 | 2.28 | 1.83 |
|  | IQR | 1.01–4.16 | 2.58–12.25 | 1.50–10.20 | 1.33–12.53 | 0.79–10.39 | 0.81–13.64 |

Abbreviations:‾x, mean; s, standard deviation. M, median; IQR, interquartile range.
