# Supplementary material for: Elevated Circulating IL-10 Producing Breg, but Not Regulatory B Cell Levels, Restrain Antibody-Mediated Rejection After Kidney Transplantation
Source: Front Immunol. 2021 Jan 28;11:627496. doi: 10.3389/fimmu.2020.627496 (PMC7877339; doi:10.3389/fimmu.2020.627496)
Supplement: Supplementary file 4 [file Table_4.docx]

**Supplementary Table 4: The data of four subpopulations of circulating Bregs at each time point in the healthy (n=9) patients.**

| ratio | statistic | day 0 | day 1 | day 7 | day 14 |
| --- | --- | --- | --- | --- | --- |
| CD19^+^CD24^+^CD27^+^  /CD19^+^ (%) | ‾x  s | 19.37  5.41 | 12.68  5.42 | 16.13  7.91 | 17.19  5.60 |
|  | M  IQR | 16.40  14.35–24.95 | 11.00  7.74–17.40 | 13.80  10.75–18.25 | 15.50  15.20–18.45 |
| CD19^+^CD24^+^CD38^+^  /CD19^+^ (%) | ‾x  s | 23.48  12.96 | 26.39  11.55 | 29.58  11.79 | 32.39  9.53 |
|  | M  IQR | 19.60  10.11–35.75 | 26.50  14.30–37.55 | 29.30  21.75–37.40 | 28.00  25.35–38.40 |
| CD19^+^CD24^+^CD27^+^IL-10^+^  /CD19^+^ (%) | ‾x  s | 3.45  4.37 | 3.32  2.79 | 1.01  0.82 | 1.47  0.96 |
|  | M  IQR | 1.21  0.71–5.71 | 2.80  0.92–5.86 | 1.06  0.26–1.48 | 0.98  0.63–2.46 |
| CD19^+^CD24^+^CD38^+^IL-10^+^  /CD19^+^ (%) | ‾x  s | 5.52  5.92 | 5.00  4.27 | 2.37  1.62 | 2.87  1.66 |
|  | M  IQR | 1.89  1.05–11.19 | 3.28  1.83–8.19 | 1.91  0.96–3.45 | 2.17  1.38–4.20 |

Abbreviations:‾x, mean; s, standard deviation. M, median; IQR, interquartile range.
